# Factors associated with hopelessness, depression and anxiety in the Honduran-Central America population during the COVID-19 pandemic

**DOI:** 10.3389/fpsyt.2023.1116881

**Published:** 2023-03-01

**Authors:** Eleonora Espinoza-Turcios, René Mauricio Gonzales-Romero, Carlos Sosa-Mendoza, Manuel Sierra-Santos, Henry Noel Castro-Ramos, Lysien Ivania Zambrano, José Armada, Christian R. Mejía

**Affiliations:** ^1^Scientific Research Unit (UIC), Faculty of Medical Sciences (FCM), Institute for Research in Medical Sciences and Right to Health (ICIMEDES), National Autonomous University of Honduras (UNAH), Tegucigalpa, Honduras; ^2^Honduran Institute for the Prevention of Alcoholism, Drug Addiction and Drug Dependency (IHADFA), Tegucigalpa, Honduras; ^3^Faculty of Business Sciences, Universidad Continental, Huancayo, Peru; ^4^Translational Medicine Research Centre, Universidad Norbert Wiener, Lima, Peru

**Keywords:** anxiety, depression, hopelessness, Honduras, mental health, Central America

## Abstract

**Introduction:**

The mental health of the population has been affected by COVID-19, reporting in many populations higher levels of depressive, anxious and stress symptoms, however, in Honduras there are no studies showing the impact of COVID-19 on the mental health of the population.

**Aim:**

To determine the factors associated with hopelessness, depression and anxiety in times of COVID-19 in the Honduran population.

**Methods:**

Cross-sectional analytical study. Three scales were used, Beck for hopelessness, Hamilton for depression and anxiety, through interviews with the population attending different levels of health care throughout the country. Descriptive and analytical statistics were obtained. For hopelessness, the Beck Hopelessness Scale was used; for depression and anxiety, the Hamilton Scale was used.

**Results:**

Of the 8,125 participants, the population in general showed results of hopelessness 14.9%, depression 12.9% and anxiety 1.2%, there was less hopelessness among women (*p* = 0.004), university students (*p* < 0.001), but greater among those who had a family member deceased by COVID-19 (*p* < 0.001), among those who had diabetes mellitus (DM) (*p* < 0.001), history of mental illness (*p* < 0.001) or had consumed drugs in the last 6 months (*p* < 0.001). There was more depression among those with arterial hypertension (*p* = 0.002), obesity (*p* = 0.019), DM (*p* = 0.004), history of mental illness (*p* < 0.001) or had consumed drugs in the last 6 months (*p* < 0.001). There was more anxiety among those infected with COVID-19 (*p* = 0.023), according to having a family member deceased by COVID-19 (*p* = 0.045) and in those with a history of mental illness (*p* < 0.001).

**Discussion:**

The Honduran population presented important differences in the socio-pathological, according to the loss of family members or having previous illnesses, this according to the presentation of problems in the mental sphere.

**Conclusion:**

We found factors associated with hopelessness, we found factors associated with hopelessness, depression and anxiety in times of COVID-19 in the Honduran population.

## Introduction

The World Health Organization (WHO) defines mental health as a state of well-being, where the person realizes his or her capabilities and is able to cope with the normal stresses of life, to work productively and to contribute to his or her community, being a state of internal dynamic equilibrium in which people can apply universal values to society, preserving basic skills, social skills, abilities to cope with life problems, to recognize, understand and express one’s own feelings (1, 2), although it is also known that mental health problems increase in situations of disasters, wars or global problems, where there is greater fear, uncertainty and stigmatization; directly impacting public health (3).

It is known that before the pandemic, depressive and anxiety mental disorders were the leading cause of the global health-related burden (4). In a study showing the prevalences of psychological consequences by COVID-19, symptoms of Post-Traumatic Stress Disorders was 33%, anxiety 28%, stress 27% and depression 22% (5). A systematic review and a meta-analysis at this time the highest levels of depression and anxiety were reported in Africa and the Americas region, respectively (6). But then came the issues of confinement, economic problems, disruption of social, academic and routine activities, which together increased psychological disorders (7). For example, in China, which was the country where the pandemic originated, 54% of psychological impact was observed, 17% of depressive symptoms, 29% of anxious symptoms and 8% of stress; all between moderate and severe (8). Following that, a meta-analysis found that mental health symptoms in adults varied between regions, where Africa had the highest prevalence rate of depression (45%), followed by South Asia (34%) and Latin America (32%); at the same time, South Asia was the highest in anxiety (41%), followed by Africa (37%) and Latin America (32%) (9). And these symptoms increased in some specific populations, such as those working in the health sector, where multiple reports show increased problems of depressive symptoms, anxiety, insomnia and stress (10).

However, this has been studied more in developed countries and others that had a great affectation, but countries like Honduras have not been the exception, having reported before the pandemic 39% of depression and 29% of anxiety; this in the rural area of Francisco Morazán and Olancho. This is related to a study in a multi-center population with chronic diseases, where Honduras had 30% depression (11, 12). Then came all the problems generated by economic instability, confinement, lack of work, the feeling of loneliness, among many others; having reported in 2020 that the “most common negative emotions were: helplessness, despair, fear, anxiety, nostalgia, restlessness and uncertainty” (13). For this reason, the objective was to determine the factors associated with hopelessness, depression and anxiety in times of COVID-19 in the population of Honduras.

## Methods

The Scientific Research Unit (UIC) of the Faculty of Medical Sciences (FMC)–UNAH, designed a strategy to implement national research on priority health problems in low-resource settings. In addition, the Honduran Institute for the Prevention of Alcoholism, Drug Addiction and Drug Dependence (IHADFA) and SESAL of Honduras also collaborated. The surveyors were 168 eighth year medical students (MSS), who received scholarships/support from the Ministry of Health (SESAL) to be trained in basic topics of practical learning methodology, applied research methods and others that would serve them during the research and the survey.

After training, the surveyors were distributed throughout the country; this distribution was carried out in hospitals and primary care units during the period from September–October 2021 to March–April 2022. Therefore, the population that was accessed was the one that went to these public health institutions, being mainly populations of the middle and lower social classes and in urban and semi-urban areas.

To determine whether the number of respondents was sufficient, statistical power was calculated for the crossover of the three dependent variables versus each of the independent variables, where almost all the crossovers were above 95%, except for the crossovers of hopelessness versus being infected by COVID-19 (69%) or having consumed alcohol in the last 6 months (67%); of depression versus sex (41%), educational level (52%); as well as, anxiety versus sex (34%), educational level (63%), obesity (63%) or having consumed alcohol (11%) or drugs (57%) in the last 6 months.

The study design was cross-sectional analytic and multi-center, with a non-random sample by convenience included 8,137 patients who were ≥ 18 years old, who were health care workers of the institutions where the survey was conducted and who identified themselves as residents of the catchment areas in hospitals, urban and semi-urban primary health care clinics. Twelve respondents were excluded because they were aged 12–17 years.

Before starting the project, ethical care was ensured, and therefore, approval was requested from the FCM Biomedical Research Ethics Committee (CEIB), approval code: 056–2021. In addition, as part of their training, the MSS were given an online course on Good Clinical Practices The Global Health Network.^1^

For the survey process, verbal consent was obtained from each participant, which was recorded in the electronic survey designed and uploaded to the Microsoft Forms platform. This form of survey was chosen due to multiple factors: In the period of patient enrollment, there were still a regular number of positive cases, so the virtual survey system helped to have less contact between the respondent and the surveyor. In addition, this system allowed respondents to feel more at ease knowing that they could not be identified, which helped them to give more reliable answers. Finally, this generated a better way of storing the surveys, centralizing the information, debugging and quality control from a single central location and saving physical material, the latter to achieve a smaller carbon footprint.

Beck Hopelessness Scale (14) is a self-application instrument designed to measure the degree of this condition in adolescents and adults, which consists of 20 items with true or false response options. The most appropriate cut-off point is 8 positive responses, so that a score equal to or higher than 8 indicates a high degree of hopelessness (15). It has been validated in Colombia (16), Mexico Quiñones, Méndez, Castañeda, 2019 (17).

The German psychiatrist Max Hamilton in 1960 described for the first time the depression scale (18), which in its original version consisted of 21 items with 3 and 5 ordinal response options, later a reduced version was made with 17 items, which is the one recommended by the National Institute of Mental Health of the United States (19). The scale assesses the severity of depressive symptoms during the week prior to the interview. The total scale score ranges from 0 points (no depressive symptoms) to 66 (severe depressive symptoms). In our study we used the scale validated for Spain (20). The Hamilton Depression Scale became and remains the “gold standard” tool for assessing the severity of depression (21).

The Hamilton Anxiety Scale is a self-administered scale of 14 items that evaluates the patient’s degree of anxiety (22). It is applied in a semi-structured form, where the severity of the symptoms is evaluated using 5 ordinal response options (0: absence of the symptom, up to 4: very severe or disabling symptom). The total score of the instrument, which is obtained by the sum of the partial scores of the 14 items, can range from 0 points (absence of anxiety) to 56 (maximum degree of anxiety) (23). In a study in Mexico, using the Beck II scale for depression and the Hamilton anxiety scale in 177 high school students during the pandemic, showed the presence of depression indicators (48.0%) and presented major anxiety, 89.8 and 32.2% showed the presence of suicidal thoughts (24) Similarly, traumatology and orthopedics residents had a high prevalence of depression and anxiety using both Hamilton scales (25).

In addition to these tests for the measurement of the dependent variables, the following questions were also asked: Gender (male or female), age (taken quantitatively), educational level (high school or less versus college or higher), whether they had been infected by COVID-19 (yes or no), whether a family member died from COVID-19 (yes or no), whether they had arterial hypertension (yes or no), obesity (yes or no), diabetes mellitus (yes or no), diagnosis of previous mental illness (yes or no), whether they had consumed alcohol or drugs in the last 6 months (yes or no).

### Statistical analysis

After the data collection process was completed, the variables were cleaned and coded, with this the database was generated. The data were analyzed with Stata V16.0 software (license acquired by the group’s statistician). First a table was generated with the description of the variables, where frequencies and percentages were used for the categorical variables, then measures of central tendency and dispersion were obtained for age. For the analytical statistics, crude prevalence ratios (cPR), adjusted prevalence ratios (aPR), 95% confidence intervals (95%CI) and *p*-values were found, all with the generalized linear models (Poisson family, log link function and models for robust variances). Before performing the multivariate analysis, a series of steps were carried out, including the generation of a univariate table to describe the population, followed by a bivariate analysis in which each of the three outcome’s (hopelessness, depression and anxiety) was crossed with the independent variables (sex, age, academic grade, having been infected by COVID-19, whether a family member died from COVID-19, whether he/she suffered from hypertension, obesity, diabetes mellitus, etc., depression and anxiety) versus the independent variables (sex, age, academic grade, having been infected by COVID-19, if a family member died from COVID-19, if they suffered from arterial hypertension, obesity, diabetes mellitus, mental illness, had consumed alcohol or drugs in the last 6 months); for the independent variables to be included in the multivariate model, they had to obtain a *p* value < 0.05 (statistical criterion). In all cases, *p*-values greater than 0.05 were considered statistically significant, this being the criterion for a variable to move from the bivariate model to the multivariate model.

## Results

Of the 8,125 respondents, 64.9% were women, the median age was 34 years (interquartile range: 26–47 years), 69.9% had high school or less, 62.4% had not yet been infected by COVID-19, 18.9% had a family member who died from COVID-19, 20.4% had AHT, 15.9% obesity, 9.3% diabetes mellitus, 2.3% history of mental illness, 28.5% alcohol, and 2.6% drugs in the past 6 months (respectively) (Table 1).

**Table 1:** General characteristics of the surveyed population of Honduras in the COVID-19 pandemic, period 2021–2022, (*n* = 8,125).

Variable	Frequency	Percentage (%)
Gender		
Male	2,851	35.1
Female	5,273	64.9
Age (years completed)		
Mean and standard deviation	37,1	13.3
Median and interquartile range	34	26–47
Education level		
High school or less	5,678	69.9
University or more	2,446	30.1
Infected with COVID-19		
No	5,065	62.4
Yes	3,059	37.6
Family member died from COVID-19		
No	6,586	81.1
Yes	1,538	18.9
Arterial hypertension		
No	6,464	79.6
Yes	1,660	20.4
Obesity		
No	6,835	84.1
Yes	1,289	15.9
Diabetes mellitus		
No	7,372	90.7
Yes	752	9.3
Mental illness		
No	7,935	97.7
Yes	189	2.3
Alcohol in last 6 months		
No	5,809	71.5
Yes	2,315	28.5
Drugs in last 6 months		
No	7,911	97.4
Yes	213	2.6

In the first multivariate model, there was less hopelessness among women (aPR: 0.79; 95%CI: 0.67–0.93; value of *p* = 0.004) and among those with university level (aPR: 0.32; 95%CI: 0.25–0.41; value of *p*<0.001), on the contrary, there was more hopelessness among those who had a family member died from COVID-19 (aPR 1.37; 95%CI: 1.15–1.64; value of *p* < 0.001), among those with diabetes mellitus (aPR 1.81; 95%CI: 1.46–2.25; value of *p* < 0.001), history of mental illness (aPR: 3.30; 95%CI: 2.49–4.38; value of *p* < 0.001) or had used drugs in the last 6 months (aPR: 2.30; 95%CI: 1.67–3.15; value of *p* < 0.001); adjusted for three variables (Table 2).

**Table 2:** Factors associated with hopelessness in Honduran residents in the COVID-19 pandemic, in the period 2021–2022, (*n* = 8,125).

Variable	Hopelessness		Type of analytical statistics	
	No *n* (%)	Yes *n* (%)	Bivariate	Multivariate
Gender				
Male	2,616 (91.8)	235 (8.2)	Comparison cat.	Comparison cat.
Female	4,922 (93.3)	351 (6.7)	0.81 (0.69–0.95) 0.008	0.79 (0.67–0.93) 0.004
Age (years completed)	34 (26–47)	37 (26–51)	1.01 (1.01–1.02) <0.001	0.99 (0.99–1.01) 0.554
Education level				
High school or less	5,163 (90.9)	515 (9.1)	Comparison cat.	Comparison cat.
University or more	2,375 (97.1)	71 (2.9)	0.32 (0.25–0.41) <0.001	0.32 (0.25–0.41) <0.001
Infected with COVID-19				
No	4,682 (92.4)	383 (7.6)	Comparison cat.	Did not enter the model
Yes	2,856 (93.4)	203 (6.6)	0.88 (0.74–1.03) 0.119	Did not enter the model
Family member died from COVID-19				
No	6,145 (93.3)	441 (6.7)	Comparison cat.	Comparison cat.
Yes	1,393 (90.6)	145 (9.4)	1.41 (1.18–1.68) <0.001	1.37 (1.15–1.64) <0.001
Arterial hypertension				
No	6,051 (93.6)	413 (6.4)	Comparison cat.	Comparison cat.
Yes	1,487 (89.6)	173 (10.4)	1.63 (1.38–1.93) <0.001	1.20 (0.97–1.48) 0.088
Obesity				
No	6,373 (93.2)	462 (6.8)	Comparison cat.	Comparison cat.
Yes	1,165 (90.4)	124 (9.6)	1.42 (1.18–1.72) <0.001	1.18 (0.97–1.44) 0.097
Diabetes mellitus				
No	6,896 (93.5)	476 (6.5)	Comparison cat.	Comparison cat.
Yes	642 (85.4)	110 (14.6)	2.27 (1.87–2.75) <0.001	1.81 (1.46–2.25) <0.001
Mental illness				
No	7,388 (93.1)	547 (6.9)	Comparison cat.	Comparison cat.
Yes	150 (79.4)	39 (20.6)	2.99 (2.24–4.00) <0.001	3.30 (2.49–4.38) <0.001
Alcohol in last 6 months				
No	5,406 (93.1)	403 (6.9)	Comparison cat.	Did not enter the model
Yes	2,132 (92.1)	183 (7.9)	1.14 (0.96–1.35) 0.128	Did not enter the model
Drugs in last 6 months				
No	7,359 (93.0)	552 (7.0)	Comparison cat.	Comparison cat.
Yes	179 (84.0)	34 (16.0)	2.29 (1.66–3.15) <0.001	2.30 (1.67–3.15) <0.001

*Variable taken as quantitative. For analytical statistics, prevalence ratios (left of parentheses), 95% confidence intervals (within parentheses) and p values (right of parentheses), obtained with the generalized linear models (Poisson family, log link function and models for robust variances) are shown.

In the second multivariate model, there was more depression among those with AHT (aPR: 1.35; 95%CI: 1.11–1.64; value of *p* = 0.002), obesity (aPR: 1.28; 95%CI: 1.04–1.57; value of *p* = 0.019), among those with diabetes mellitus (aPR: 1.43; 95%CI: 1.13–1.82; value of *p* = 0.004), history of mental illness (aPR: 4.73; 95%CI: 3.77–5.93; value of *p* < 0.001) or had used drugs in the last 6 months (aPR: 2.08; 95%CI: 1.47–2.94; value of *p* < 0.001); adjusted for three variables (Table 3).

**Table 3:** Factors associated with depression in Honduran residents in the COVID-19 pandemic, in the period 2021–2022, (n = 8,125).

Variable	Depression		Type of analytical statistics	
	No *n* (%)	Yes *n* (%)	Bivariate	Multivariate
Gender				
Male	2,642 (92.7)	209 (7.3)	Comparison cat.	Did not enter the model
Female	925 (93.4)	348 (6.6)	0.90 (0.76–1.06) 0.213	Did not enter the model
Age (years completed)	34 (26–47)	35 (26–49)	1.05 (0.99–1.01) 0.126	Did not enter the model
Education level				
High school or less	5,276 (92.9)	402 (7.1)	Comparison cat.	Did not enter the model
University or more	2,291 (93.7)	155 (6.3)	0.90 (0.75–1.07) 0.225	Did not enter the model
Infected with COVID-19				
No	4,751 (93.8)	314 (6.2)	Comparison cat.	Comparison cat.
Yes	2,816 (92.1)	243 (7.9)	1.28 (1.09–1.51) 0.003	1.15 (0.98–1.36) 0.094
Family member died from COVID-19				
No	6,155 (93.5)	431 (6.5)	Comparison cat.	Comparison cat.
Yes	1,412 (91.8)	126 (8.2)	1.25 (1.03–1.51) 0.021	1.10 (0.91–1.34) 0.323
Arterial hypertension				
No	6,067 (93.9)	397 (6.1)	Comparison cat.	Comparison cat.
Si	1,500 (90.4)	160 (9.6)	1.57 (1.32–1.87) <0.001	1.35 (1.11–1.64) 0.002
Obesity				
No	6,405 (93.7)	430 (6.3)	Comparison cat.	Comparison cat.
Yes	1,162 (90.2)	127 (9.8)	1.57 (1.30–1.89) <0.001	1.28 (1.04–1.57) 0.019
Diabetes mellitus				
No	6,900 (93.6)	472 (6.4)	Comparison cat.	Comparison cat.
Yes	667 (88.7)	85 (11.3)	1.77 (1.42–2.20) <0.001	1.43 (1.13–1.82) 0.004
Mental illness				
No	7,439 (93.7)	496 (6.3)	Comparison cat.	Comparison cat.
Yes	128 (67.7)	61 (32.8)	5.16 (4.13–6.46) <0.001	4.73 (3.77–5.93) <0.001
Alcohol in last 6 months				
No	5,443 (93.7)	366 (6.3)	Comparison cat.	Comparison cat.
Yes	2,124 (91.8)	191 (8.2)	1.31 (1.11–1.55) 0.002	1.15 (0.96–1.38) 0.134
Drugs in last 6 months				
No	7,386 (93.4)	525 (6.6)	Comparison cat.	Comparison cat.
Yes	181 (85.0)	32 (15.0)	2.26 (1.63–3.15) <0.001	2.08 (1.47–2.94) <0.001

*Variable taken as quantitative. For analytical statistics, prevalence ratios (left of parentheses), 95% confidence intervals (within parentheses) and p-values (right of parentheses), obtained with the generalized linear models (Poisson family, log link function and models for robust variances) are shown.

In the third multivariate model, there was more anxiety among those who were infected with COVID-19 (aPR: 1.97; 95%CI: 1.10–3.53; value of *p* = 0.023), in those who had a family member deceased by COVID-19 (aPR: 1.83; 95%CI: 1.01–3.31; value of *p* = 0.045) and in those with a history of mental illness (aPR: 11.97; 95%CI: 6.09–23.50 value of *p*<0.001; Table 4).

**Table 4:** Factors associated with anxiety in residents of Honduras in the COVID-19 pandemic, in the period 2021–2022, (*n* = 8,125).

Variable	Anxiety		Type of analytical statistics	
	No *n* (%)	Yes *n* (%)	Bivariate	Multivariate
Gender				
Male	2,832 (99.3)	19 (0.7)	Comparison cat.	Did not enter the model
Female	5,246 (99.5)	27 (0.5)	0.77 (0.43–1.38) 0.377	Did not enter the model
Age (years completed)	34 (26–47)	41 (26–50)	1.02 (0.99–1.04) 0.122	Did not enter the model
Education level				
High school or less	5,651 (99.5)	27 (0.5)	Comparison cat.	Did not enter the model
University or more	2,427 (99.2)	19 (0.8)	1.63 (0.91–2.93) 0.100	Did not enter the model
Infected with COVID-19				
No	5,046 (99.6)	19 (0.4)	Comparison cat.	Comparison cat.
Yes	3,032 (99.1)	27 (0.9)	2.35 (1.31–4.22) 0.004	1.97 (1.10–3.53) 0.023
Family member died from COVID-19				
No	6,556 (99.5)	30 (0.5)	Comparison cat.	Comparison cat.
Yes	1,522 (98.9)	16 (1.1)	2.28 (1.25–4.18) 0.007	1.83 (1.01–3.31) 0.045
Arterial hypertension				
No	6,435 (99.6)	29 (0.4)	Comparison cat.	Comparison cat.
Si	1,643 (99.9)	17 (1.0)	2.28 (1.26–4.14) 0.007	2.04 (1.13–3.72) 0.019
Obesity				
No	6,799 (99.5)	36 (0.5)	Comparison cat.	Did not enter the model
Yes	1,279 (99.2)	10 (0.8)	1.47 (0.73–2.96) 0.277	Did not enter the model
Diabetes mellitus				
No	7,334 (99.5)	38 (0.5)	Comparison cat.	Did not enter the model
Yes	744 (98.9)	8 (1.1)	2.06 (0.97–4.40) 0.061	Did not enter the model
Mental illness				
No	7,900 (99.6)	35 (0.4)	Comparison cat.	Comparison cat.
Yes	178 (94.2)	11 (5.8)	13.20 (6.81–25.58) <0.001	11.97 (6.09–23.50) <0.001
Alcohol in last 6 months				
No	5,777 (99.5)	32 (0.5)	Comparison cat.	Did not enter the model
Yes	2,301 (99.4)	14 (0.6)	1.10 (0.59–2.05) 0.770	Did not enter the model
Drugs in last 6 months				
No	7,867 (99.4)	44 (0.6)	Comparison cat.	Did not enter the model
Yes	211 (99.1)	2 (0.9)	1.69 (0.41–6.92) 0.467	Did not enter the model

*Variable taken as quantitative. For analytical statistics, prevalence ratios (left of parentheses), 95% confidence intervals (inside parentheses) and p-values (right of parentheses), obtained with generalized linear models (Poisson family, log link function and models for robust variances) are shown.

## Discussion

The aim of the study was to evaluate the levels of hopelessness, depression and anxiety in the Honduran population ≥ 18 years during the COVID-19 pandemic. This being important because Honduras does not have national studies on the mental health situation of the general population, there are specific studies that establish prevalence in certain communities, Paz-Fonseca et al. (26) found a prevalence for anxiety of 20.5%, depression of 13.2%. Women in the family were the most affected and the prevalence of alcoholism was 6.2% (26).

In 33 rural communities in Honduras, it was found that 35% of those interviewed were women, the most frequent disorders were: Major Depressive Episode 24%, Agoraphobia 9.3% and Social Phobia 6%, in the 923 men surveyed: Alcohol Dependence 16.1%, Major Depressive Episode 13.2% and Social Phobia 6%, Generalized Anxiety Disorder 5% (27).

In Latin America the pooled prevalence of anxiety, depression, distress and insomnia was 35, 35, 32 and 35%, respectively (28).

The results of our study were 65% women, young adults, middle school education, a large majority had not been infected by COVID-19, among the chronic non-communicable diseases, HT, obesity and DM were reported as risk factors for COVID-19, it is noteworthy that a high percentage had not been contaminated, but 19% had a family member who died from COVID-19. Robinson (29) reported that there was a small increase in mental health symptoms shortly after the outbreak of the COVID-19 pandemic that declined and was comparable to pre-pandemic levels in mid-2020 among most population subgroups and symptom types, mental health symptoms during March–April 2020. Compared with measures of anxiety and general mental health, increases in symptoms of depression and mood disorders tended to be greater and remained significantly elevated in May–July (29).

According to the sex variable, women were more affected than men in their mental health, different from what was reported in our study, men were more affected in the three pathologies, although it does coincide with the literature where women were more affected than men for major depressive disorder, anxiety disorders and younger age groups were more affected than older groups (4).

There are more vulnerable groups in case of extreme situations such as catastrophes, pandemics, health workers are a group highly exposed to anxiety, depression, stress and other mental health problems (8), the recent pandemic by COVID-19, has had adverse effects on mental health (4) in the general population and in specific populations: women and health workers, who are at particular risk of suffering a deterioration in mental wellbeing (30).

Large-scale meta-analysis of the prevalence of mental health problems during the early COVID-19 pandemic, women and persons with COVID-19 infection had higher rates in almost all outcomes; college students/young adults of anxiety, depression, sleep problems, suicidal ideation; adults with fear and post-traumatic symptoms. Anxiety, depression, and posttraumatic symptoms were more prevalent in low/middle-income countries, and sleep problems in high-income countries (31).

In a study in Turkey, levels of hopelessness and anxiety were higher in health professionals than in non-health workers. Levels of hopelessness in nurses were higher than in physicians, and levels of anxiety were higher than in physicians and other health care workers. Levels of anxiety and hopelessness were higher in women who lived with a high-risk person in the household during the pandemic, who had difficulty caring for their children, and who had decreased income. Anxiety levels are an important predictor of hopelessness. Increased levels of anxiety explained 28.9% of the increase in levels of hopelessness; in our study of the three pathologies studied, anxiety had the lowest level and was reported more in men than in women (32). In the systematic review and meta-analysis by Arora T, the prevalence of psychological outcomes was similar in healthcare workers and the general population 34% (24–44) and 33% (27–40) (5).

According to Gan-Yi Wang, the COVID-19 pandemic has been affecting people’s psychosocial health and well-being through several complex pathways, more than one-third reported a worsening experience of hopelessness and loneliness, with more than two-fifths reporting worsening depression during the pandemic compared to before the outbreak. Several socioeconomic and lifestyle factors were found to be associated, marital status, household income, smoking, alcohol consumption and existing chronic conditions of the participants, 44.8% expressed feeling depressed, 34.8% more hopeless and 32.5% lonely during the pandemic. The percentage of all three indicators was higher among women than among men, being married was associated with lower odds of loneliness among men. Loneliness was negatively associated with smoking and positively associated with alcohol consumption (33).

Rodriguez et al. studied the risk factors associated with depression, anxiety in COVID-19 pandemic, where their prevalences for depression and anxiety were 31 and 42%, there was also a prevalence of youth within the age range 18–23, most were women, it was observed that it prevailed in single adults or young people living with their parents, since with an abrupt confinement, the economically active population was the most affected, at the same time causing unemployment and those who were single without having an emotional support to accompany them in this situation (34, 35).

We found a lower prevalence of hopelessness among women and among those with a university level; on the contrary, there was more hopelessness among those who had a family member who had died from COVID-19, those with diabetes mellitus (DM), and had a history of mental illness or had consumed drugs in the last 6 months. In China, a meta-analysis on comorbidities and COVID-19 in 2021, AHT, DM-2, cardiovascular diseases, chronic kidney diseases have been among the most frequent comorbidities in patients with COVID-19, in another study in the same country, it was found in a cohort of 99 patients, 51% patients had chronic diseases and of them 40.4% were CVD and Cardiovascular, 12% DM, 11% digestive system diseases and these comorbidities increased the risk of mortality (36, 37).

The prevalence of depressive disorders in diabetics ranges from 10.0 to 15.0% (38, 39), which is practically twice as high as the prevalence in non-diabetics. For Turkey burnout, hopelessness, and fear of COVID19, it shows the hopelessness scores of working in departments that contained a high risk of COVID-19 infection, having a history of COVID-19 infection, working with insensitive supervisors, feeling at risk of COVID-19 due to work, being exposed to excessive workload, working for low wages, not having enough time for oneself or one’s own family, and feeling uncomfortable about putting loved ones at risk of COVID-19 (40).

Vai and Col, found greater hopelessness, being the antecedent to have a mental illness, according to the literature people with any mental disorder had a higher probability of being hospitalized because of COVID-19 (41). Our study reported more depression among those who had AHT, obesity, those with DM, history of mental illness or had used drugs in the last 6 months, depression is a common mental disorder, it is estimated that 5% of adults suffer from depression, it is one of the leading causes of disability worldwide, more women are affected by depression than men (42, 43).

Vai in 2021, describes that the main factors associated with depression were sex, previous psychiatric history, psychopathology at 1-month follow-up, and systemic inflammation during the acute phase, whereas age was only a potential factor and severity of acute COVID-19 was not. In fact, female sex, a previous psychiatric diagnosis, and psychopathology at 1-month follow-up were moderators of depression in the post-COVID-19 syndrome (41).

A systematic review and meta-analysis, from January 1985 to August 2021, included 44 studies. The prevalence of depression was significantly higher in people with type 1 diabetes or type 2 diabetes compared with those without diabetes. There was no association between study effect size and mean age or sex. The findings did not differ significantly between methods of depression assessment. Depression was higher in patients with diabetes in studies conducted in specialty care compared with those in community or primary care and in low- and middle-income countries compared with countries with high-income economies (44).

According to WHO in 2016, 39% of adults aged 18 years or older (39% of men and 40% of women) were overweight, 13% of the global adult population (11% of men and 15% of women) were obese (43). Overweight/obesity and depression are highly concurrent conditions with shared pathophysiology, as well as social and economic determinants (45). Anxiety and depression are as strongly predictive of future poor physical health as obesity and smoking (46).

People with obesity and diabetes are at increased risk for significant symptoms of depression (47), physical activity, nutrition, and eating behaviors are associated with physical and mental health, hence the importance of establishing and strengthening healthy lifestyle habits in this target population depression and obesity are complex and chronic (48). During COVID-19, the prevalence of alcohol use disorders increased from 25.1% before confinement to 38.3% during confinement in England (49).

We found in our study that there was more anxiety among those who were infected with COVID-19, in those who had a family member who died from COVID-19, and in those who had a history of mental illness. Anxiety disorders were more prevalent among those aged 30 to 39 years (18.2%) (50). Fear of the unknown increases anxiety levels in healthy individuals as well as in those with preexisting mental health conditions (51).

In a meta-analysis including 194 studies, the overall prevalence of anxiety was 35.1%. The prevalence in low- and middle-income countries was similar compared with high-income countries. One in three adults lived with anxiety disorder during the COVID-19 pandemic worldwide (52).

In the case of DM, in Pakistan, 22.6% of diabetic participants presented mild depression and 2.6% presented moderate depression. In Latin America, 1,508 patients were evaluated in Mexico, of which 30.7% presented comorbidities such as AHT or DM, in addition 18% presented depression and 27.8% anxiety, showing that the relationship between comorbidity and psychological impact is maintained in different parts of the world with populations suffering from non-communicable pathological diseases and psychiatric illnesses, causing a psychosocial impact (53, 54).

Neuropsychiatric symptoms were evaluated with a meta-analysis after COVID-19, the most prevalent neuropsychiatric symptom was sleep disturbance followed by fatigue, objective cognitive impairment, anxiety, and post-traumatic stress. Cognitive impairment, anxiety, post-traumatic symptoms, and depression are also common in the first 6 months (55). COMET-G at the beginning of the pandemic found rates of anxiety 25%, depression 28% in the general population, while a second study reported 31.9 and 33.7% anxiety and depression, respectively (12).

The uncertainties associated with a new virus, the risk of staff becoming infected, a changing and challenging work environment, the potential personal impact of the virus, and the concerns associated with caring for patients and their families put additional pressure on staff (56). Studies examining the mental health impact of providing front-line medical care during viral outbreaks showed that healthcare workers often have high levels of anxiety, depression, and post-traumatic stress disorder, both during and after outbreaks We identified a wide number of risk factors such as younger age and female gender, and social factors such as lack of social support, social rejection, or isolation and stigmatization. Occupational factors involved working in a high-risk environment (frontline staff), specific occupational roles (e.g., nurse), and having lower levels of specialized training, preparation, and work experience (50).

A meta-analysis has identified, on a large scale and worldwide, the prevalence of mental health symptoms, it was found that healthcare workers exposed to COVID-19 had a significant prevalence rate of anxiety, depression, acute stress, insomnia, post-traumatic symptoms, and burnout, are a vulnerable population during the COVID-19 pandemic, being more prone to mental health impairment than the general population. These findings suggest that the mental health impairment of healthcare workers is not due to measures of general confinement, social distance, and pandemic preoccupation, but to the particularities of the healthcare professions and their conditions during the pandemic (57).

Data suggest that the pandemic and associated public health and social measures (PHSM) have led to a global increase in mental health problems, including, across the board, depression and anxiety. Persons with pre-existing mental disorders are also at increased risk of severe illness and death from COVID-19 and should be considered an at-risk group when diagnosed with the infection (58) Yuan K, reported that participants with a history of mental disorders displayed over three times higher risk for depression and anxiety (59).

In the year 2020 in Latin America and the Caribbean, the SARS-COV pandemic has led to increases in unemployment, poverty, food insecurity, domestic violence, and child abuse at the same time that worsening mental health conditions were reported in the same area (58), in the Human Development Report 2020, Honduras presented an HDI of 0.634, which places it in position 132 out of 189, being the most worrisome situation as a result of the COVID-19 pandemic (60).

The COVID-19 pandemic negatively affects mental health in a unique way across all population subgroups. Our results inform tailored preventive strategies and interventions to mitigate current, future, and transgenerational adverse mental health from the COVID-19 pandemic (31).

### Limitations

Our database is predominantly composed of individuals of the female gender. One limitation was not being able to access remote or rural locations, this in part was due to poor Wi-Fi/internet signal in those remote or very distant locations; This limitation means that the results cannot be extrapolated to the rural population of Honduras, which is confirmed by the fact that there was no probability sampling, so the sample is important but still cannot represent the entire population of Honduras, but it could be an important situational analysis of patients attending hospitals or health care centers. Another limitation was that some of the crosstabs did not have adequate statistical power, so these crosstabs should be considered purely exploratory. The approach was carried out in primary health care (PHC), and for this reason the diagnoses were not confirmed by a physician specializing in psychiatry. For all these reasons, it should be remembered that extrapolations to other populations should be made with caution in the descriptive results, but the associations are important because they come from a large group of citizens attending public health facilities.

## Conclusion

Our study shows that the most reported variable was hopelessness, followed by depression and very low anxiety. Men in Honduras had the highest levels of hopelessness, depression and anxiety, and by the end of the pandemic a high percentage of the general population had not yet been infected by COVID-19. A considerable percentage of the participants had suffered the loss of a family member, which negatively influences the deplorable mental health of the population in times of COVID-19.

Chronic non-communicable diseases such as HTN, obesity and DM were the most prevalent for all three pathologies, as was the history of mental illness. It is important for countries to be concerned about the mental health of the population, which prior to the pandemic was already a major public health problem worldwide and which was exacerbated as a result of COVID-19.

The pandemic came to change our way of living, to change our habits and, faced with the uncertainty of the unknown, made us vulnerable at the expense of finding the solution in a vaccine that would stop the virus and mitigate the panic generated worldwide.

## Data availability statement

The raw data supporting the conclusions of this article will be made available by the authors, without undue reservation.

## Ethics statement

The studies involving human participants were reviewed and approved by Comité de Ética en Investigación Biomédica (CEIB) de la Facultad de Cencías Medicas-UNAH. The ethics committee waived the requirement of written informed consent for participation.

## Author contributions

EE-T, CS-M, RG-R, and MS-S: study design. RG-R and HC-R: data collection. CM, LZ, and JA: data analysis. EE-T, CS-M, CM, LZ, JA, RG-R, and HC-R: writing. All authors contributed to the article and approved the submitted version.

## Funding

The current article processing charges (publication fees) were funded by the Facultad de Ciencias Médicas (FCM) (2–03–01-01), Universidad Nacional Autónoma de Honduras (UNAH), Tegucigalpa, MDC, Honduras, Central America (granted to EE-T).

## Conflict of interest

The authors declare that the research was conducted in the absence of any commercial or financial relationships that could be construed as a potential conflict of interest.

## Publisher’s note

All claims expressed in this article are solely those of the authors and do not necessarily represent those of their affiliated organizations, or those of the publisher, the editors and the reviewers. Any product that may be evaluated in this article, or claim that may be made by its manufacturer, is not guaranteed or endorsed by the publisher.

## Footnotes

^1^ www.tghn.org
